# Cannabinoids: A New Perspective on Epileptogenesis and Seizure Treatment in Early Life in Basic and Clinical Studies

**DOI:** 10.3389/fnbeh.2020.610484

**Published:** 2021-01-12

**Authors:** Angélica Vega-García, Iris Feria-Romero, Anais García-Juárez, Ana Ch. Munguia-Madera, Alexia V. Montes-Aparicio, Esli Zequeida-Muñoz, Estefany Garcia-Albavera, Sandra Orozco-Suárez

**Affiliations:** ^1^Departamento de Fisiología, Facultad de Medicina, Universidad Nacional Autónoma de México, Ciudad de México, Mexico; ^2^Unidad de Investigación Médica en Enfermedades Neurológicas, Hospital de Especialidades, “Dr. Bernardo Sepúlveda”, Centro Médico Nacional Siglo XXI, Instituto Mexicano del Seguro Social, IMSS, Ciudad de México, Mexico; ^3^División de Ciencias Biológicas y Ambientales, Centro Universitario de Ciencias Biológicas y Agropecuarias, Universidad de Guadalajara, Guadalajara, Mexico; ^4^Facultad de Medicina, Universidad Autónoma de Guerrero, Acapulco, Mexico; ^5^Facultad de Biología, Universidad Autónoma del Estado de Morelos, Cuernavaca, Mexico

**Keywords:** cannabinoids, epileptogenesis, neurodevelopment, neuroprotection, anti-inflammatory, pharmacokinetics

## Abstract

Neural hyperexcitability in the event of damage during early life, such as hyperthermia, hypoxia, traumatic brain injury, status epilepticus, or a pre-existing neuroinflammatory condition, can promote the process of epileptogenesis, which is defined as the sequence of events that converts a normal circuit into a hyperexcitable circuit and represents the time that occurs between the damaging event and the development of spontaneous seizure activity or the establishment of epilepsy. Epilepsy is the most common neurological disease in the world, characterized by the presence of seizures recurring without apparent provocation. Cannabidiol (CBD), a phytocannabinoid derived from the subspecies *Cannabis sativa* (*CS*), is the most studied active ingredient and is currently studied as a therapeutic strategy: it is an anticonvulsant mainly used in children with catastrophic epileptic syndromes and has also been reported to have anti-inflammatory and antioxidant effects, supporting it as a therapeutic strategy with neuroprotective potential. However, the mechanisms by which CBD exerts these effects are not entirely known, and the few studies on acute and chronic models in immature animals have provided contradictory results. Thus, it is difficult to evaluate the therapeutic profile of CBD, as well as the involvement of the endocannabinoid system in epileptogenesis in the immature brain. Therefore, this review focuses on the collection of scientific data in animal models, as well as information from clinical studies on the effects of cannabinoids on epileptogenesis and their anticonvulsant and adverse effects in early life.

## Introduction

Seizure disorders are common during childhood; they are causes of morbidity (Glass et al., 2018), and a large percentage of them have a poor response to current first-line anticonvulsant drugs (ADs; Glass et al., 2012). In the postnatal neurodevelopmental period, neuronal excitability is predominantly mediated by the glutamatergic and GABAergic activity system, and it promotes the processes of growth, plasticity, synaptogenesis, and organization of neural networks essential for the adult stage (Ben-Ari, 2002; Ben-Ari and Holmes, 2006; Rakhade and Jensen, 2009). Thus, the immature brain is highly susceptible to developing neuronal hyperexcitability under pathological conditions such as hyperthermia, hypoxia-ischemia, traumatic brain injury (TBI), or a pre-existing neuroinflammatory condition that in turn facilitates the development of seizure activity and the establishment of *status epilepticus* (*SE*; Pitkänen et al., 2015; Suchomelova et al., 2015).

Additionally, the early exposure to many ADs, is a significant risk for brain development such as PB, DFH, and valproate, and has been related to developmental disorders (Bittigau et al., 2002; Forcelli et al., 2011; Kaushal et al., 2016; Al-Muhtasib et al., 2018). Furthermore, clinical evidence indicates that gestational exposure to ADs can also lead to deficits in cognitive function (Meador et al., 2012). For this reason, the need to identify new drugs, Cannabidiol (CBD), and its propyl cannabidivarin (CBDV) analog, has aroused interest in the treatment of epilepsy in early life. While CBD was recently approved for the treatment of refractory childhood epilepsies (Abu-Sawwa et al., 2020), little is known about the efficacy and safety of compounds derived from *Cannabis sativa* (*CS*) in the early stage of development (Rosenberg et al., 2017). Here we address this issue through a systematic evaluation of the cannabinoid literature investigating multiple therapeutic targets, some of which were tested in early developmental seizure models, including data from our laboratory as well as clinical evidence.

### Cannabinoids

*CS* is an herbaceous plant native to central Asia that is widely distributed in a variety of habitats and altitudes and is a unique species of its kind. Some authors recognize *C. ruderalis* and *C. indica* as separate species or subspecies of *CS*; however, a monospecific criterion has been adopted in many of the nontaxonomic publications, since all the groups of plants that have been included within the genus are interfertile and their morphological diversity shows a diffuse and continuous pattern (Etienne, 2014). This plant has been known for its medicinal and textile uses that date back to more than 5,000 years ago. Currently, a total of 545 constituents of cannabis have been identified, of which 104 are phytocannabinoids classified into 11 types: (−)-Δ^9^-trans-tetrahydrocannabinol (Δ^9^ THC) type, (−)-Δ^8^-trans-tetrahydrocannabinol (Δ^8^-THC) type, cannabigerol (CBG) type, cannabichrome (CBC) type, cannabidiol (CBD) type, cannabidiol (CBND) type, cannabielsoin (CBE) type, cannabicyclol (CBL) type, cannabinol (CBN) type, cannabitriol (CBT) type, and miscellaneous cannabinoid type. Additional compounds include flavonoids, steroids, phenanthrenes, xanthones, and sugars, among others (Pertwee, 2008; El Sohly et al., 2016). Phytocannabinoids are terpenophenolic products that exhibit a 21- to a 22-carbon skeleton, some of which are breakdown products of other cannabinoids. The predominant compounds in the plant are tetrahydrocannabinol acid (THCA), cannabidiolic acid (CBDA), and cannabinolic acid (CBNA), followed by cannabigerolic acid (CBGA), cannabichromenic acid (CBCA), and cannabinodiolic acid (CBNDA). Although THCA is the most important compound in drug-type cannabis and CBDA in fiber-type cannabis, it is also worth noting that CBCA predominates in young plants and decreases with maturation (Andre et al., 2016). Nevertheless, *CS* is not the only natural source of cannabinoids, as, in the Radula and Helichrysum gender, the presence of cannabinoid-type terpenophenolics has also been reported; however, little is known about these compounds (Mahmoud and Waseem, 2014; Andre et al., 2016). Currently, the pharmacological actions of the psychotropic cannabinoids Δ^9^-THC, Δ^8^-THC, CBN, and Δ^9^-THCV and the non-psychotropic phytocannabinoids CBC, CBD, CBDA, CBG, THCA, THCVA, CBGA, CBDV and CBGV have been well documented. Also, pharmacological actions of the nonphytocannabinoid component (E)-β-caryophyllene, which is a sesquiterpene, have also been described (Mechoulam and Gaoni, 1965; Pertwee, 2008; Mechoulam and Parker, 2013; Pertwee and Cascio, 2014).

### Endocannabinoid System

The endocannabinoid system (eCBs) was discovered by Mechoulam and Gaoni (1965), who isolated the active compound (Δ^9^-THC); in 1990, they discovered the binding site of this cannabinoid, oriented their search for additional receptors, and described the abundant CB1 receptor (CB1R) in the central nervous system (CNS) with a similar density to the GABA and glutamate receptors. Years later, the CB2 receptor (CB2R), abundant in tissues of the immune system, was described, followed by the functioning of its endogenous ligands, the endocannabinoids 2-arachidonoyl glycerol (2-AG) and *N*-arachidonoyl ethanolamide or anandamide (AEA; Andre et al., 2016). Similarly, how the endocannabinoids 2-AG and anandamide or AEA are synthesized from fatty acids from the remodeling of the cell lipid membrane and are produced according to the individual’s bodily demands was described shortly thereafter (Di Marzo and Piscitelli, 2015). Anandamide is produced by the action of the enzyme *N*-acyltransferase (NAT), which produces *N*-arachidonoyl phosphatidylethanolamine (NArPE). Phospholipase D generates a family of compounds, the arachidonoyl glycerol ethanolamine (FAE) family, which includes 2-AG and anandamide. When these compounds are released, their degradation occurs through metabolism by enzymatic hydrolysis, where fatty acid amide hydrolase (FAAH) intervenes in the degradation of anandamide and monoacylglycerol lipase (MAGL). Finally, 2-AG is metabolized into glycerol and arachidonic acid during postsynaptic cell recapture. Anandamide and 2-AG can also bind to plasma-circulating albumin and have distant effects (Patel et al., 2017). NAT is regulated by calcium and cAMP cyclic adenosine monophosphate and is selectively stimulated by cellular depolarization or by the action of the metabotropic receptors for glutamic acid, dopamine, or acetylcholine (Lu and Mackie, 2016; Patel et al., 2017).

**Figure 1:**
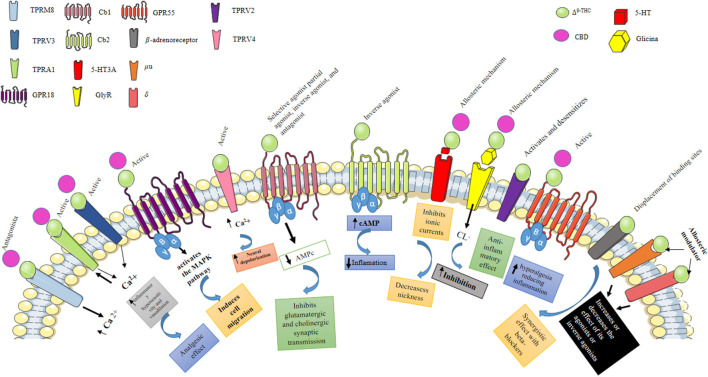
Pharmacological effects of Δ^9^-THC and CBD. Δ^9^-THC is the main psychoactive component of *C. sativa*, which can behave as a selective agonist, partial agonist, inverse agonist, and antagonist of the Cb1 receptor, while when activating the Cb2 receptor it behaves as an inverse agonist. Activation of the Cb1 and Cb2 receptors stimulates GTPγS binding to cell membranes and inhibits cyclic AMP production. Also, Δ^9^-THC can inhibit 5HT^3A^ receptor-mediated currents induced by 5-hydroxytryptamine (5HT); antagonizing receptor activation, possibly through an allosteric mechanism, by this same mechanism, Δ^9^-THC and CBD can enhance the activation of GlyR expressed in central tegmental area (ATV) neurons. Additionally, Δ^9^-THC activates the TRPV3 and TRPV4 receptors, which are nonselective calcium-permeable cation channels that, when activated, raise intracellular Ca^2+^ and consequently cause neuronal depolarization. Transient receptor potential (TRP) channels are a group of membrane proteins involved in the transduction of a large number of stimuli. Unlike Δ^9^-THC, CBD does not activate CB1 and CB2 receptors, which likely accounts for its lack of psychotropic activity. However, CBD interacts with many other, nonendocannabinoid signaling systems. It is a “multi-target” drug. At low micromolar to submicromolar concentrations, CBD is a blocker of the equilibrative nucleoside transporter (ENT), the orphan G-protein-coupled receptor GPR55, and the TRP of melastatin type 8 (TRPM8) channel. At higher micromolar concentrations, CBD activates the TRP of vanilloid type 1 (TRPV1) and 2 (TRPV2) channels while also inhibiting cellular uptake and fatty acid amide hydrolase—catalyzed degradation of anandamide.

The abovementioned endocannabinoids act as agonists of these receptors and are G protein-coupled to the endocannabinoid CB1R and CB2R. G proteins inhibit adenylate cyclase activation by reducing cAMP concentrations and interfering with the activity of cAMP-dependent protein kinase (PKA). The same G protein activates mitogen activation-dependent protein kinase (MAPK); both PKA and MAPK are involved in the selective expression of genes. The activation of the CB1R and CB2R inhibits L-, N-, P-, and Q-type voltage-dependent Ca^2+^ channels, which reduces the entry of Ca^2+^ and stimulates the endogenous K+ rectifier channels at the neuronal level by allowing the release of ions, resulting in hyperpolarization. Interactions with other receptors, such as GPR55, GPCR, and TRPV1 (in this case acting as an antagonist), have also been described, in which the endocannabinoids act as neuromodulators (Castillo et al., 2012; Alexandre et al., 2020; Figure 1). The CB1R is expressed throughout the CNS, particularly in the hippocampus on mossy fibers of the granule cells of the dentate gyrus (DG), wherein the mature stage, eCBs regulate the efficiency of inhibitory synapses during periods of sustained depolarization at the postsynaptic level through increased intracellular Ca^2+^, which activates the synthesis of endocannabinoids, mainly in pyramidal neurons of the hippocampus (Herkenham et al., 1990, 1991; Katona et al., 1999, 2006). These eCB are fat-soluble, which allows their diffusion through the plasma membrane as well as the synaptic cleft in a retrograde manner towards the neighboring presynaptic terminals, favoring the inhibitory activity mediated by the GABAergic system (Katona et al., 1999, 2006; Kawamura et al., 2006; Monory et al., 2006).

### Endocannabinoid System in Postnatal Neurodevelopment

It has been shown that during critical periods of neurodevelopment, eCBs participate in the maturation of corticolimbic circuits, where excitatory neuronal activity is essential for the processes of synaptogenesis and neuronal plasticity (Harkany et al., 2008), directed by the glutaminergic system, which constitutes most synapses; likewise, ion channels and glutamate transporters and receptors are expressed at levels that promote the activity of excitatory networks (Sanchez et al., 2001; Jensen, 2009). In rodents, during the first week, from 0 to 7 post natal (PN) days, a period analogous to a 36-week, premature human, the GABAergic system participates in excitatory neurotransmission, mediated by an increase in the K^+^/Cl cotransporter NKCC1 and downregulation of KCC2. The neuronal depolarization is generated by intracellular Cl^−^ increases and activates L-type voltage-sensitive Ca^2+^ channels (L-VSCC) and NMDARs, which increases the flux of intracellular Ca^2+^ and activates signaling cascades directed at intracellular trophic activity, neuritic growth, neural network formation, and synaptogenesis (Ben-Ari, 2002, 2014; Galanopoulou, 2008). During this period of neurodevelopment, CB1R is located in the terminals of mossy fibers in the CA3 subfield of the hippocampus, mainly in GABAergic and cholecystokinin (CCK) circuits, reaching a maximum concentration at the day of 4 PN, where CB1Rs are coupled to G proteins. In these terminals, CB1Rs suppresses the release of GABA, reducing the excitatory activity of GABA and exerting a role as a regulator of excitatory neuronal activity important for the development of neuronal circuits (Bernard et al., 2005; Fernández-Ruiz et al., 2005).

The eCBs modulate neuronal activity by regulating retrograde signaling, which participates in short-term synaptic plasticity, also known as suppression of depolarization-induced inhibition (DSI) and excitation (DSE). It is estimated that eCB levels, specifically those of AEA during development, determine the direction of synaptic plasticity (Kreitzer and Regehr, 2001; Castillo et al., 2012). From PN day 8, GABA begins to exert its inhibitory activity due to the increase in expression of the KCC2 cotransporter, which decreases intracellular Cl^−^ levels, favoring neuronal hyperpolarization and the inhibitory activity of eCBs (Ben-Ari, 2002, 2014; Galanopoulou, 2008). Then, from PN days 10–14 in the rat, which corresponds to the first 36 months of human life, GluRs are regulated by glia, and ionotropic glutamate receptors (iGluRs) are linked to ion channels that allow the flow of sodium (Na^+^), potassium (K^+^) and Ca^2+^ ions to different degrees depending on the receptor subunit. The main GluRs are NMDARs, AMPARs, and KARs (Benítez-Diaz et al., 2003; Herlenius and Lagercrantz, 2004; Rakhade and Jensen, 2009). During this period, NMDARs are expressed differently in the brain, with an increase in the expression of NR2B and NR3A subunits, which are not very sensitive to Mg^+2^ ions, which increases the activation of the receptor and increases intracellular Ca^2+^ flux, thereby promoting neuronal hyperexcitability and long-term potentiation (LTP; Ben-Ari, 2002, 2014; Qu et al., 2003; Coulter, 2006).

The NMDARs distributed in the immature brain are mainly found in the hippocampus at the postsynaptic level, but some are also present at the presynaptic level and in astrocytes (Lee et al., 2010; Szczurowska and Mareš, 2013; Skowrońska et al., 2019). The glutamate receptors AMPARs and KARs are permeable to Ca^2+^ ions when GluR2 expression is relatively low or absent (Romjin et al., 1991; Tyzio et al., 1999; Jensen, 2009); KARs are also permeable to Ca^2+^ when the GluR5 and GluR6 subunits are absent. Additionally, metabotropic glutamate receptors (mGluRs) are coupled to a G protein (GTP) and mediate slow synaptic responses (Jensen, 2009; Ben-Ari, 2014), which favor activation of CB1R expressed in glutamatergic neurons to modulate excitatory and inhibitory neurotransmission. This process is essential in the remodeling of inhibitory circuits during adolescence, a period from PN day 30 in rodents, where the regulation of glutamatergic and GABAergic activity are of vital importance for the formation, maturity, and elimination of synapses, mainly at the level of the prefrontal cortex (PFC), which has greater activity compared with the adult stage, a period from PN day 60 in rodents (Clancy et al., 2001; Auvin and Dupuis, 2014; Dow-Edwards and Silva, 2017). During adolescence, eCBs participate in the formation of neural networks in the prefrontal cortex and maintain the interaction between the amygdala, hippocampus, and hypothalamus, which is responsible for cognitive and emotional development since endocannabinoids are responsible for the normal response to stress and the regulation of neuronal excitation and inhibition (Schonhofen et al., 2018; Cheung et al., 2019).

The expression of CB2R in cells of the immune system at the peripheral level has been reported in CD4^+^, CD8^+^ T and B lymphocytes, natural killer cells, monocytes, and polymorphonuclear neutrophils (Atwood and Mackie, 2010). CB2R is also expressed in the microglia, which during postnatal neurodevelopment maintain amoeboid forms that allow them to actively participate in the phagocytosis of apoptotic cell debris, in the induction of apoptosis in other cells during the formation of functional neural circuits, and the removal of residual myelin, as well as in the formation and expansion of neural networks that imply the pruning of synapses at the level of the cerebral cortex, hippocampus, cerebellum, and amygdala (Berdyshev, 2000; Bessis et al., 2007; Atwood and Mackie, 2010; Onaivi et al., 2012). In adult rats, however, the expression of CB2R decreases as the microglia become inactive (Stella, 2004; Fernández-Ruiz et al., 2005; Caiati et al., 2012). Additionally, the expression of CB2R has been reported in glutamatergic and GABAergic neurons in the DG and CA1 subfield of the hippocampus at the level of the pyramidal stratum and stratum radiatum to a lesser degree than CB1R, which is observed mainly in the cerebral cortex, hippocampus, amygdala, and cerebellum (Brusco et al., 2008; Onaivi et al., 2012).

Astrocytes and microglia are responsible for the surveillance and modulation of the immune response and are the main cytokine producers in the CNS (Baud and Saint-Faust, 2019). Here, CB2R has been reported to participate as a neuromodulator of the neuroactive molecules ON, glutamate, PGs, and neurotrophins in the glia and vascular endothelium, which participate in neurodevelopmental processes, homeostatic mechanisms such as sleep, and activation of signaling pathways involved in neuronal plasticity and synaptogenesis; however, the mechanisms responsible for its participation in eCBs during postnatal neurodevelopmental processes are not yet entirely clear (Devinsky et al., 2013; Schonhofen et al., 2018; Cheung et al., 2019).

### Endocannabinoid System in Epileptogenesis in the Immature Brain

**Figure 2:**
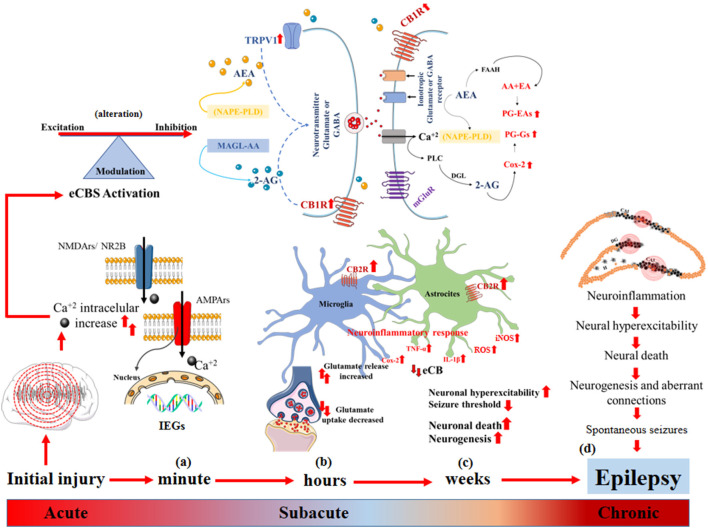
Epileptogenesis and endocannabinoid system (eCBs). **(A)** Acute changes: increased intracellular Ca^2+^ flux, induction of early genes (IEGs) that alter synaptic function, decreased threshold to neuronal hyperexcitability, alteration of eCBs that modulates the balance between excitatory and inhibitory neurotransmission. **(B)** Sub-acute changes: synthesis of endocannabinoids (eCB) mediated by the increase in intracellular Ca^2+^, neuronal hyperexcitability and the demand for membrane phospholipids diacylglycerol lipase (DAGL) and *N*-acyl phosphatidyl ethanolamine-hydrolyzing phospholipase D (NAPE-PLD) to form 2-AG and AEA, respectively, and their degradation after the dissociation of CB1R and the activation of TRPTV1 by AEA, which triggers greater glutamate release and increases in intracellular Ca^2+^. The eCBs degrade rapidly, and 2-AG and AEA are catabolized by the enzymes monoacylglycerol lipase (MAGL) and fatty acid amide hydrolase (FAAH), respectively, which generate AA, PGs-Eas, PG-Gs, and Cox-2 to increase convulsive susceptibility, activation of astrocytes and microglia and increases in CB2R that promote the release of pro-inflammatory proteins IL1-β, Cox-2, TNF-α and iNOS, generating neuroinflammation, neuronal hyperexcitability, and neuronal death. **(C,D)** Chronic changes: neuronal death, the perpetuation of the neuroinflammatory response and dysregulation of eCBs that promote neurogenesis, formation of aberrant connections, expression of spontaneous seizures, and epilepsy.

The term epileptogenesis is defined as a sequence of events that convert a normal circuit into a hyperexcitable circuit (Pitkänen et al., 2015). Epileptogenesis refers to the development of tissue capable of generating spontaneous seizures, which result in an epileptic condition or in the progression of established epilepsy (Pitkänen and Engel, 2014), whereby it is a continuous and multifactorial process in which the eCBs participate in the modulation of neuronal activity, neuronal migration, axonal growth and guidance, synaptic plasticity, and the neuroinflammatory response (Schonhofen et al., 2018; Cheung et al., 2019). It has been established that damage to eCB signaling in the early stages of neurodevelopment can favor the process of epileptogenesis and the establishment of epilepsy in later stages (Figure 2).

During the latent period, the participation of eCBs has been described in different cascades of events defined as acute, subacute, and chronic (Kadam et al., 2010). The induction of early genes (IEGs), including Fos, Jun, Egr4, Egr1, Homer 1, Nurr77, and Arc, occurs during acute changes as a result of intense and repeated synaptic activity during seizure activity, which promotes increased intracellular Ca^2+^ flux by NMDARs (Herdegen and Leah, 1998). Upregulation of IEGs can pathologically modulate synaptic function, lowering the threshold to neuronal hyperexcitability (Rakhade and Jensen, 2009). The levels of intracellular Ca^2+^ immediately alters synaptic efficiency mechanisms, such as increasing the postsynaptic density and dendritic spines, grouping NMDARs and AMPARs, and increasing glutamate synthesis (Haglid et al., 1994; Sanchez et al., 2001), where the participation of eCBs plays an important pathophysiological role in modifying excitatory and inhibitory synaptic neurotransmission in the brain (Rosenberg et al., 2017). During postnatal neurodevelopment, CB1R located in presynaptic cells, mainly in the granule cells of the hippocampus, regulates excitatory activity; additionally, endocannabinoids can also act through interactions with other types of receptors, mainly on G protein-coupled receptors, such as GPRSS and the Transient receptor potential (TRP) cation channel subfamily V member 1" or “vanilloid receptor 1” (TRPV1), which participate in neuronal hyperexcitability, favoring intracellular Ca^2+^ flux (Bhaskaran and Smith, 2010; Schonhofen et al., 2018; Cheung et al., 2019).

As we have indicated, the synthesis of endocannabinoids is mediated by an increase in intracellular Ca^2+^ generated by neuronal hyperexcitability and the demand for membrane phospholipid diacylglycerol lipase (DAGL) and for glutamate and acetylcholine (Wallace et al., 2003; Rosenberg et al., 2017). The eCBs passively diffuse through presynaptic cells in a retrograde manner to bind orthosterically to and activate CBR1 acylphosphatidyl ethanolamine-hydrolyzing phospholipase D (NAPE-PLD) to form 2-AG and AEA, in the same way, endocannabinoids are also synthesized from the activation of metabotropic receptors and thus inhibit the release of glutamate or GABA, a process defined as depolarization-induced suppression of inhibition (DSI) or excitation (DSE), respectively (Armstrong et al., 2009). In models of febrile seizures at PN day 10, alterations to these DSI and DSE mechanisms have been reported, which are estimated to interfere with the maturation of the GABAergic system and persist in later stages through the generation of hyperexcitable circuits (Bernard et al., 2005).

Similarly, it has been reported that eCB signaling has an important role in epileptogenesis, because, after the dissociation from CB1R, eCB 2-AG and AEA are catabolized by the enzymes monoacylglycerol lipase (MAGL) and alpha-beta hydrolase domain containing 6 (CABHD6) or through fatty acid amide hydrolase (FAAH), respectively, and activation of TRPV1 by AEA can trigger increased glutamate release after intracellular Ca^2+^ concentrations are increased (Wallace et al., 2003; Rosenberg et al., 2017). This process activates calcineurin (PPP3C or serine-threonine protein phosphatase 2B), which, during intense seizure activity, activates calcium-calmodulin-phosphatase to promote the dephosphorylation and subsequent endocytosis of GABA_A_ (Blair et al., 2004). These phenomena reduce the inhibitory potential and lead to the gradual loss of GABAergic inhibitory networks (Kurz et al., 2001; Blair et al., 2004; Rakhade and Jensen, 2009; Semple et al., 2020). In the immature brain, calcineurin activation increases the phosphorylation of Kv2.1 ion channels (also known as KCNB1) and promotes their expression in the postsynaptic membrane, allowing prolonged neuronal depolarization (Kurz et al., 2001; Rakhade and Jensen, 2009).

Similarly, protein kinase C, which is dependent on protein kinase type II and calcium-calmodulin-phosphatase, increases within minutes after the induction of *SE* in immature rats, which allows an increase in the phosphorylation of serine 831 of GluR1 and serine 880 of GluR2. This process promotes the endocytosis of GluR2 and increases the permeability to Ca^2+^ in AMPARs, which generates an increase in seizure susceptibility in later or adult stages (Rice and De Lorenzo, 1998; Sanchez et al., 2001; Rakhade and Jensen, 2009). Additionally, it has been reported that astrocyte activation, immediate to neuronal damage, increases extracellular K^+^ concentrations, which in turn facilitates continuous neuronal hyperexcitability, a triggering factor for epileptogenesis (Jabs et al., 1997; Jensen, 2009).

Subacute changes in the latent period are established from hours to days after neuronal damage (Figure 2), in which a rapid increase occurs in NGF and BDNF, which together with Trk alter the modulation of the maturation of the KCC2 cotransporter, promoting GABA-mediated inhibitory activity mainly in DG granular cells, which in turn alters the modulating function of eCBs on the GABAergic system to favor long-term neuronal hyperexcitability (Chen et al., 2003, 2007). However, the cellular mechanisms by which CB1R and CB2R act on short- and long-term changes in the seizure threshold are still unknown, particularly in mesiotemporal structures consisting of mainly the hippocampus and the cerebral cortex in the immature brain (Chen et al., 2007).

The neuroinflammatory response activated by microglia and astrocytes, the main response responsible for the synthesis and release of pro-inflammatory cytokines such as IL1-β, activates the IL-1/TLR signaling pathway, where an increase in pro-inflammatory proteins occur. These proteins include Cox-2, an enzyme that catalyzes the production of PGs from AA and increases seizure susceptibility (Chen et al., 2001, 2007; Jiang et al., 2004; Feng et al., 2016), and TNF-α, which generates an increase in the expression of AMPAR and a decrease in GluR2 through the expression of TNF-R1 on the neuronal membrane surface, which in turn causes increased permeability of immature neurons to Ca^2+^ (Beattie et al., 2002; Stellwagen et al., 2005). The activity of TNF-α has been shown to result in the endocytosis of GABA_A_ receptors, mainly their β 2/3 subunits, in the pyramidal neurons of CA1 and interneurons of the DG, promoting neuronal excitation (Wang et al., 2000; Balosso et al., 2005; Stellwagen et al., 2005; Shimada et al., 2014) and leading to neurodegeneration and neuronal death in the CA1 and CA3 regions of the immature hippocampus (Rizzi et al., 2003; Kawaguchi et al., 2005).

In the same way, the rapid increase in Cox-2 mRNA expression after *SE* induced by KA itself induces neurodegeneration and neuronal death in the immature hippocampus, mainly the CA1 and CA3 subfields, at 12 and 24 h post *SE* (De Simoni et al., 2000; Rizzi et al., 2003; Kawaguchi et al., 2005). This finding supports that the neuroinflammatory response precedes neuronal damage (Ravizza et al., 2005; Joseph and Levine, 2006), and this neuronal damage is associated with an increase in Cox-2 and the subsequent production of PGs and ROS, activation of the oxidative stress mechanism, and mitochondrial dysfunction (Gobbo and O’Mara, 2004); thus, it has been reported that the eCBs participate as a substrate of the Cox-2 enzyme for the synthesis of PGs (Nomura et al., 2011; Ruhaak et al., 2011). Similarly, experimental models of *SE* induced by KA and Li-Pilo in PN 12 day rats have reported a rapid increase in ROS and mitochondrial markers of oxidative damage, including NT-3, 4-HNE and carbonylated proteins, as well as the formation of free radicals 24 h post-*SE*, which are responsible for neurodegeneration in the subfields of CA1, CA3 and DG of the hippocampus, cerebral cortex and thalamus (Patel and Li, 2003; Folbergrová et al., 2010).

The contribution of mitochondrial dysfunction on complex I of the respiratory chain NADH (Cock, 2002; Kudin et al., 2002) favor the development of neurodegenerative disorders, such as epilepsy, due to the excessive release of glutamate, which causes an alteration in the reuptake mechanisms of this neurotransmitter and an increase in intracellular Ca^2+^ due to the saturation of the regulatory mechanisms mediated by the Na^+^/Ca^2+^ exchanger and the Ca^2+^ buffer proteins (Tretter et al., 2004).This causes the mitochondria to capture the excess Ca^2+^ in the mitochondrial matrix, which induces the depolarization of the membrane by the partial inhibition of the chemiosmotic potential and by the accumulation of positive charges in the mitochondrial matrix (Tretter et al., 2004). This sustained overload produces an irreversible depolarization through the activation of the mitochondrial transition pore, a pathway through which Ca^2+^ returns to the cytosol, causing the collapse of the mitochondrial chemiosmotic potential and a reduction in the synthesis of ATP (Murchison and Griffith, 2000). The decrease in ATP generates metabolic dysfunction, ROS production, activation of proteases, phospholipases, iNOS, and endonucleases, and inhibition of protein synthesis. Furthermore, NO can act as a retrograde messenger, enhancing the excitotoxic effect of glutamate and increasing its release from presynaptic terminals (Lorigados et al., 2013). Likewise, it has been reported that complex I dysfunction may also be the result of an increase in carbonylated proteins during the subacute phase post-*SE* induced by Li-Pilo and that this alteration persists up to 5 weeks, a period in which spontaneous convulsive activity is observed (Folbergrová et al., 2010).

Likewise, Cox-2 and TNF-α act directly on receptors and ion channels and indirectly by modulating extracellular glutamate reuptake systems *via* the reduction of selective transporters to glutamate GLT-1, mediated by astrocytes, mainly in CA1 and DG of the hippocampus. Additionally, the activation of iNOS, which promotes the synthesis of NO, increases the release of glutamate and substance P, which preserves the activation of cytokines together with the increase in the affinity of NMDARs and AMPARs, which supports the finding that the activation of the neuroinflammatory response by microglia and astrocytes increases the vulnerability of the immature hippocampus to neuronal hyperexcitability and that these changes depend on the age of development (De Simoni et al., 2000; Rizzi et al., 2003; Viviani et al., 2003; Ravizza et al., 2005; Stellwagen et al., 2005; Bessis et al., 2007; Chen et al., 2013). The participation of CB2R, which is overexpressed by microglia in the neuroinflammatory response in response to neuronal damage and the infiltration of cells of the immune system into the brain parenchyma (Sagredo et al., 2009; Bouchard et al., 2012), induces chronic CB2R activation, which increases excitatory neurotransmission (Li and Kim, 2015) and decreases inhibitory neurotransmission (Morgan et al., 2009). The CB2R, which are mostly expressed in microglia, have been reported to participate in the neuroinflammatory response in experimental models in rats, where they increase 2-AG and CB1R in the hippocampus after *SE* (Wallace et al., 2003); however, the increase in 2-AG in the seizure model in mice shows a decrease in seizure activity (Sugaya et al., 2016).

Chronic changes (Figure 2) can be observed over 2–12 weeks in rodents and from months to years in humans. It has been reported that during the first 2 weeks of life, the resistance to excitotoxic damage in the immature brain is relative since the amount of Ca^2+^ that enters a pyramidal neuron is directly related to the age of PN development, where in the first 3 days of life, glutamate minimally increases intracellular Ca^2+^. Conversely, between PN days 10 and 25, intracellular Ca^2+^ increases markedly due to recurrent seizure activity, leading to neuronal death in the CA1 subfield of the hippocampus, the area most vulnerable to excitotoxic damage, due to an increase in NMDARs and AMPARs (Kubová et al., 2001, 2012; Kubová and Mareš, 2013). Additionally, an alteration in the immature glutamatergic system that results in the overexpression of GluR2 and its activity on the intracellular Ca^2+^ flux through AMPARs and KARs, a condition that pathologically persists during the adolescent stage mainly in the prefrontal cortex, favors the formation of hyperexcitable circuits. Similarly, prolonged positive regulation has been reported in the expression of the CB1R and in the suppression of the inhibition induced by depolarization, which can cause alterations in neuronal hyperexcitability throughout life (Chen et al., 2003; Bernard et al., 2005).

### Anticonvulsant and Neuroprotective Effect of CBD on Experimental Models of *Status Epilepticus* and Epilepsy

It has been reported that CBD has a low affinity for CB1R and CB2, and at high levels, it can act as an indirect CB1R antagonist as evaluated by a wide range of experimental models of seizures and epilepsy in adult rats. The evaluation of high doses of CBD, up to 300 mg/kg i.p., in the hippocampal kindling model reveals a reduction in amplitude of the discharge and an increase in the after-discharge threshold (ADT; Turkanis et al., 1979; Ghovanloo et al., 2018). However, in the lamotrigine drug-resistant tonsillar kindling model, CBD does not show anticonvulsant or neuroprotective effects (Klein et al., 2017). Though, in both models, potential neuroprotective effects were identified at low vs. high doses of CBD, which increased the neurotoxic effects of this active ingredient (Patra et al., 2019). Conversely, no anticonvulsant effects of repeated CBD administration have been reported (Rosenberg et al., 2017). The neuroprotective effects of CBD have been identified when administered after *SE* induced by intrahippocampal pilocarpine microinjection and in *in vitro* models in rat hippocampal slices, in which CBD decreases the amplitude and duration of the epileptiform activity induced by 4-aminopyridine (Jones et al., 2010; Franco and Perucca, 2019). However, despite the experimental existence of the anticonvulsant effects of CBD, the responsible mechanisms remain unclear, and few studies have examined its anticonvulsant effect in the immature stage (Table 1).

**Table 1:** Effect of cannabinoids in experimental models of epileptic seizures.

Compound	Type of study	Effect	Mechanism	Reference
CBD (HU-320)	*In vivo* Mouse CIA model. *In vitro* Mouse macrophages and RAW 267.7 cells.	Antiinflammatory	Inhibition of IL-1β, pro-inflammatory cytokines, and TNF-α.	Burstein (2015)
CBD	*In vivo* Hypoxia-ischemia model in newborn pigs.	Neuroprotective	Increasing eCB levels through CB2 receptors and as a 5HT_1A_ agonist.	Leo et al. (2016)
CBD	*In vivo* Electrophysiology model in female and male Wistar rats, free of Mg^2^ and 4-AP.	Anticonvulsant	Multi-electrodes in hippocampus.	Leo et al. (2016)
	*In vitro* Male Wistar Rat PTZ Seizure Model.	Anticonvulsant	Displacing the selective SR141716A receptor antagonist.	Jones et al. (2010)
CBD	*In vitro* Mouse fibroblasts and human B lymphoblastoid serum.	Antioxidant	Pathway not mediated by receptors. It antagonizes oxidative stress, and the consequent cell death induced by the retinoid anhydroretinol.	Booz (2011)
CBD	*In vivo* BCCAO model in male mice.	Neuroprotective	Decreased expression of GFAP 7.	Mori et al. (2017)
CBD	*In vivo* In RVM of rats treated with CFA.	Antiinflammatory	Regulation of the release of pro-inflammatory and anti-inflammatory cytokines.	Bouchet and Ingram (2020)
CB_2_ synthetic	*In vivo* EM Viral Model (Theiler).	Antiinflammatory	Modulation in cytokines of the IL-12 family.	Correa et al. (2007)
THC THC-A CBD CBDA	*In vitro* Human colon adenocarcinoma. HT 29 cell culture.	Antiinflammatory	Cox inhibition	Ruhaak et al. (2011)
CBD	*In vitro/in vivo* AD model.	Neuroprotective	Nitrite levels reduction.	Mecha et al. (2016)
CBD	*In vivo* HI model in newborn mice.	Neuroprotective	Decreased glutamate levels, IL-6, TNF-α, and Cox-2.	Castillo et al. (2012)
CBD	*In vivo* Ischemic damage model.	Antiinflammatory	HMGB1 decrease in microglia.	Hayakawa et al. (2009)
CBD	*In vivo* HI model in newborn Wistar rats.	Neuroprotective	Modulation of the expression of oxidative stress and inflammation.	Pazos et al. (2012)
CBD	*In vivo* Hippocampal kindling in adult rats.	Anticonvulsant	CB1R antagonism	Turkanis et al. (1979)
CBD	*In vitro* Model of SE by intrahippocampal pilocarpine.	Neuroprotective	Decreased amplitude and duration of epileptiform activity.	Jones et al. (2010)
CBD	*In vitro* Cultivo de células HEK 293A y células ST Hdh.	Anticonvulsant	Modulatory effect on neuronal hyperexcitability through GPR 55 antagonism.	Laprairie et al. (2015)
CBD	*In vivo* Human coronary artery atherosclerosis.	Antiinflammatory	Inhibition in the pro-inflammatory pathway of NF-kB, decreased nitrotyrosine, iNOS, ICAM1, and VECAM1.	Rajesh et al. (2007)
CBD	*In vivo* Rat uveitis model.	Antiinflammatory	Modulation of macrophage and microglial function. Nitrogen activating protein kinase activation.	El-Remessy et al. (2008)
CBD	Lymph node cells from mice.	Antiinflammatory	Decreased expression of IL-1β, TNFα, and MAPKs.	Malfait et al. (2000)

*CBD cannabidiol; HU-320, modified CBD structure; CIA, collagen-induced arthritis; 5HT_1a_, 5-hydroxytryptamine; 4-AP, 4-aminopiridina; PTZ, pentilenetetrazol; SR141716A, N-(piperidin-1-yl)-5-(4-chlorophenyl)-1-(2,4-dichlorophenyl)-4-methyl-1hpyrazole-3-carboxamide; BCCAO, bilateral common carotid artery, occlusion; GFAP, glial fibrillary acidic protein; RVM, rostral ventromedial medulla; CFA, complete Freund’s adjuvant; EM, esclerosis multiple; THC, tetrahydrocannabinol; THC-A, tetrahydrocannabinolic acid; CBDA, cannabidiolic acid; CBG, cannabigerol; AD, Alzheimer’s disease; HI, hypoxic-ischemic; HMGB1, high mobility group box 1; CB1R, cannabinoid type 1 receptor; SE, status epilepticus; KA, kainic acid; PN, post natal; GPR 55, G protein-coupled receptor 55; NF-KB, nuclear transcription factor kappa B; iNOS, inducible nitric oxide synthase; ICAM1, intercellular adhesion molecules; VECAM1, vascular adhesion molecules; IL-1β, interleukin-1β; TNFα, tumor necrosis factor y; MAPKs, mitogen-activated protein kinase*.

**Figure 3:**
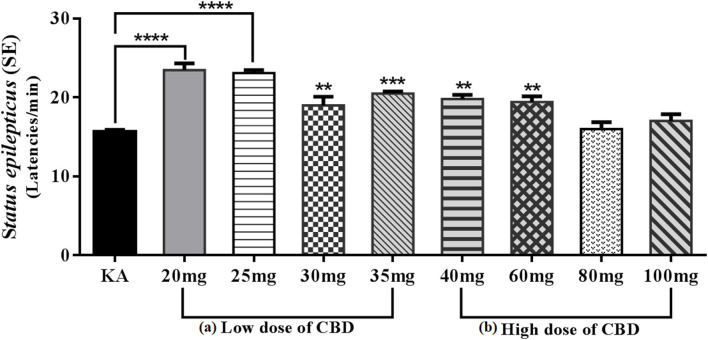
Graph showing the mean ± SE of the latencies of *Status epilepticus* (SE) induced by KA. **(A)** Low doses: the 20 and 25 mg/kg CBD groups showed an increase of SE latencies with a significant difference ****p < 0.0001 compared with the KA group. However, the 30 and 35 mg/kg groups of CBD showed a reduction in SE latencies though with a significant difference, **p < 0.01 and ***p < 0.001, respectively, compared with the KA group.** (B)** The high-dose 40 and 60 mg/kg groups of CBD showed an increase in the latencies of SE with a significant difference, **p < 0.01 compared with the KA group. The 80 and 100 mg/kg CBD groups did not show significant differences compared with the KA group. One-way ANOVA followed by Bonferroni’s *post hoc* test, *p* < 0.05 (unpublished data).

Therefore, we decided to carry out a pilot study to evaluate the anticonvulsant effect of CBD for 12 days PN on the model of *SE* induced by KA 3 mg/kg delivered *via* an intraperitoneal route, which showed that the oral route of low doses (20 and 25 mg/kg) of CBD 30 min before KA caused behavioral arrest; however, administration of higher doses (30 and 35 mg/kg) of CBD resulted in changes in motor behavior such as startles, wet dog shakes and lower limb myoclonus. During the evaluation of the anticonvulsant effect, compared with the KA group, the 20 and 25 mg/kg CBD groups showed a significant increase in *SE* initiation latency (*p* < 0.0001), while the 30 and 35 mg/kg CBD groups showed significant reductions in *SE* initiation latency (*p* < 0.01 and *p* < 0.001, respectively). Conversely, in the high-dose groups of 40, 60, 80, and 90 mg/kg CBD administered 30 min before KA, chewing, wet dog shaking, and unilateral myoclonus as well as scratching movements with the lower extremities were observed. Although CBD induced these behaviors, the anticonvulsant effect in the 40 and 60 mg/kg groups was efficient in significantly reducing the *SE* initiation latency (*p* < 0.01) relative to the KA group; however, in the 80 and 100 mg/kg of CBD groups, an increase in the severity of *SE* was observed (Figure 3). Motor behavior has been previously reported in experimental models of seizures and KA-induced SE in 10–14-day-old immature PN rats (Vega-García et al., 2020), which indicates that in SE models, an increase in the dose has opposite effects to those in adults (Jones et al., 2010; Hill et al., 2014; Klein et al., 2017). Agreeing with this, Anderson et al. (2020) in the Scn1a^+/–^ mouse model of Dravet syndrome, detected that subchronic oral administration of Δ^9^-THC or CBD alone did not affect spontaneous seizure frequency or mortality while, surprisingly, their co-administration (70 mg Δ^9^-THC + 3,500 mg CBD/kg chow) increased the severity of spontaneous seizures and overall mortality. The authors point out the detrimental outcome might simply be explained by a significant pharmacokinetic interaction between CBD and Δ^9^-THC, as plasma and brain concentrations of both Δ^9^-THC and CBD were dramatically increased, which has been demonstrated in mice and rats in other epilepsy models (Sofia et al., 1976; Chan et al., 1996).

Additionally, the anticonvulsant effect was examined in a Dravet syndrome model in voltage-gated sodium channel Nav1-knockout mice. Treatment with CBD from PN days 21–27 decreased the severity and number of seizures induced by hyperthermia as well as the number of spontaneous seizures (Kaplan et al., 2017). However, the mechanisms responsible for the anticonvulsant effect of CBD remain unclear. Previous experimental studies have reported that the anticonvulsant effect of CBD is linked to the modulating effects on neuronal hyperexcitability through the antagonism of G protein-coupled receptor 55 (GPR55), of which CBD is a negative allosteric modulator (Laprairie et al., 2015), desensitization of TRPV1 (Chen and Hackos, 2015) and inhibition of adenosine reuptake by blocking equilibrative nucleotide transporter 1 (ENT1), which increases the concentration of extracellular adenosine (Pandolfo et al., 2011). These mechanisms decrease intracellular Ca^2+^ flux and regulate neuronal hyperexcitability. These effects have also been tested in GPR55- and TRPV1-knockout mice (Klein et al., 2017; Stott et al., 2018; Franco and Perucca, 2019).

Moreover, the activity of CBD has been identified in blocking voltage-gated sodium channels and T-type Ca^2+^ channels and in the modulation of VDAC1 (Ghovanloo et al., 2018). Similarly, the interaction of CBD with voltage-gated potassium channels has been shown to have an effect on glycine α1 and α3 receptors and on the modulation of TNF-α (Gaston and Friedman, 2017; Gaston and Szafarski, 2018). It has been reported that CBD modulates the activity of the immune system in a concentration-dependent manner (Pertwee, 2008), showing that the anti-inflammatory, antioxidant and neuroprotective effects of CBD are attributed to its agonist activity on CB1R and CB2R (Ibeas et al., 2015). Also, CBD has been shown to act as an inhibitor of fatty acid amide hydrolase (FAAH), an enzyme involved in the degradation of eCB (Capasso et al., 2008). Likewise, CBD acts as a competitive inhibitor of adenosine uptake *via* ENT1 in microglia and increases exogenous adenosine, which activates the A2A receptor (Liou et al., 2008; Haskó et al., 2009); nevertheless, the mechanism of action of the A2A receptor on the modulation of the inflammatory response activated by microglia is unclear (Haskó et al., 2009; Rosenberg et al., 2017).

Pretreatment with CBD attenuates the formation of ROS and the mitochondrial damage generated by the increase in glutamate. Similarly, in models of artery sclerosis, treatment with CBD inhibits the pro-inflammatory pathway of NF-kB and decreases nitrotyrosine and iNOS, while in the endothelial cells of human coronary arteries, it decreases adhesion molecules ICAM-1 and VECAM-1. In the same way, CBD attenuates the transendothelial migration of monocytes (Rajesh et al., 2007). The antioxidant effect of CBD has been reported in a model of nephropathy induced by cisplatin, reducing ROS and generating NAPDH oxidases, iNOS and NT-3, which reduce cell death and improve kidney function (Pan et al., 2009). Therefore, it is suggested that the antioxidant effect of CBD acts at the mitochondrial level (Booz, 2011). Additionally, CBD has been reported to act as an antagonist of the GPR55 receptor, which decreases intracellular Ca^2+^ flux and modulates neuronal hyperexcitability (Rosenberg et al., 2017).

There are few reports on the neuroprotective effects of CBD in developing epilepsy models. In newborn Wistar rats that underwent IH injury (10% oxygen for 120 min after carotid artery electrocoagulation), CBD (1 mg/kg single dose, 1 mg/kg every 24 h for 72 h, or 1 mg/kg every 8 h for 72 h) modulated the expression of oxidative stress and inflammation, which reduced neuronal death and produced greater functional recovery (Pazos et al., 2012). Similarly, in a sciatic nerve section model of 2 PN day rats that received treatment with a single dose of 15 or 30 mg/kg CBD, 30% synaptic preservation was observed by immunohistochemical analysis (obtained by synaptophysin staining). The administration of CBD decreased astroglial and microglial reactions by 30 and 27%, respectively, and reduced the number of apoptotic cells mainly in the intermediate zone of the spinal cord (Pérez et al., 2013).

## Clinical Studies

### Cannabis-Derived Approved Drugs

While there are many descriptive and anecdotal reports on the benefits of cannabinoids, few cannabinoid drugs are regulated for clinical use. The most important licensed cannabis-based drugs are described herein: (a) dronabinol (Marinol^®^) was approved by the FDA on May 31, 1985, for nausea and vomiting control, associated with cancer chemotherapy that had not responded adequately to conventional antiemetic treatments. First marketed in 1986, on December 22, 1992, Marinol^®^ was approved for the relief of anorexia associated with weight loss in patients with HIV-AIDS (human immunodeficiency virus/acquired immunodeficiency syndrome). At the time of its approval, the FDA recognized that dronabinol Δ^9^-THC was considered to be the psychoactive component of marijuana. Marinol^®^ is also licensed for use in other countries, such as Canada and Germany. Generic forms of dronabinol (first approved in 2011) have now been approved in the US (Wright and Guy, 2014); (b) nabilone (Cesamet^®^ dl-3-(1,1-dimethyl heptyl)-6,6ab 7, 8, 10, 10a alpha-hexahydro-1-hydroxy-6,6-dimethyl-9H-dibenzo pyran-9-(one) is a synthetic analog of Δ^9^ THC that was developed in the 1970s long before the target receptor was identified. It has a low nanomolar affinity for the CB1 receptor and a somewhat reduced affinity for the CB2 receptor. It was initially approved by the FDA in 1985 for chemotherapy-induced nausea and vomiting that do not respond to conventional antiemetics. The drug was marketed in the United States until 2006 (Wright and Guy, 2014); (c) nabiximols (Sativex^®^) differs from the other authorized cannabinoids insofar as it comprises an extract of the cannabis plant. It is formulated as a sublingual/oromucosal spray, every 100 μl of which provides 2.7 mg of Δ^9^ THC and 2.5 mg of CBD. It was approved for the first time in Canada in 2005 under legislation that allows the conditional approval of a new drug in areas of high unmet medical need and in the presence of very promising clinical data. Its approval in the USA was delayed until the end of 2010 and early 2011 when Savitex was approved by the UK and Spain following a decentralized procedure. It is licensed as second-line therapy for the relief of spasticity in multiple sclerosis (MS); in some other countries, it may also be prescribed for the treatment of neuropathic pain in people with MS and for the treatment of cancer-related pain, where it is used as an opioid supplement (Wright and Guy, 2014); and (d) epidiolex, is the first plant-derived, purified pharmaceutical-grade CBD medication. It was approved in the USA by the FDA on June 25, 2018 (Sekar and Pack, 2019). Its approval for patients ≥2 years of age with DS or LGS markedly altered the treatment of medically refractory seizures in these disorders. Epidiolex is a CBD only component with no Δ^9^THC, and the psychoactive component of cannabis responsible for appetite stimulation and euphoria sensation (Abu-Sawwa et al., 2020; Specchio et al., 2020).

### Pharmacokinetics of Cannabinoids

To date, few clinical studies have been conducted in pediatric patients with difficult-to-control epilepsy and oral administration with cannabidiol solutions (CBD) to determine the pharmacokinetic parameters. In this review, data were obtained from a population with an age range between 0–20 years (Devinsky et al., 2018a; Wheless et al., 2019). The pharmacokinetics of cannabinoids is divided into four phases: absorption, distribution, metabolism, and excretion, but they are modified by interactions with other drugs. The pharmacokinetics of these compounds are divided into four phases: absorption, distribution, metabolism, and excretion, but they are modified by interactions with other drugs.

#### Absorption

The absorption rate of cannabinoids is determined by the route of administration of the drug and its formulation. Administration through oral formulations of any cannabinoid or food presents variable absorption and extensive first-pass hepatic metabolism. The administration of oral formulations presents a variable absorption depending on whether it is a single dose or repeated doses, as well as an extensive first-pass hepatic metabolism. The geometric mean of the time to maximum plasma concentration (*T*_max_) of cannabidiol ranged from 2 to 4 h after the administration of a single dose in a concentration range from 5–20 mg/kg without previous consumption of food. Thus, the route of administration of this pharmaceutical form has a slow absorption with a wide absorption phase. Concerning the administration of repeated doses (7 days), the *T*_max_ of cannabidiol was detected between 2–3 h in a concentration range between 10–40 mg/kg/day (Wheless et al., 2019). Sublingual administration is very similar to oral administration. Oro-mucosal or spray preparations of Δ^9^THC—CBD (1:1) allow rapid absorption, whereas higher plasma concentrations are reached compared with oral administrations, but inhaled Δ^9^ THC leads to lower concentrations in the plasma (Karschner et al., 2011; Pertwee and Cascio, 2014).

#### Distribution

Once absorbed, CBD can be detected in plasma. The geometric means of the maximum plasma concentration (*C*_max_) were 29.12, 47.19, and 103.7 ng/ml with a single dose administration of 5, 10, and 20 mg/kg, respectively. Also, an increase in the geometric means of *C*_max_ of 3.12, 2.67, and 3.03 times was observed in repeated doses of 10, 20, and 40 mg/kg for 7 days compared with the single dose. The geometric averages of the area under the plasma concentration curve at time 0–12 h [AUC (0–12 h)] were 122, 243.6, and 473.5 ng·h/ml with the administration of a single dose of 5, 10, and 20 mg/kg, respectively. Additionally, an increase of 4.15, 3.43, and 4.45 times in AUC(0-τ) was observed with the administration of repeated doses of 10, 20, and 40 mg/kg for 7 days compared with the single-dose (Wheless et al., 2019).

#### Metabolism

Both CBD and its metabolites 7-OH CBD and 7-COOH-CBD are metabolized in the liver and can induce the expression of CYP1A1, CYP1A2, CYP2C9, and CYP2D6 during prolonged periods of administration (Greene and Saunders, 1974; Bornheim et al., 1992; Watanabe et al., 2007). Pharmacokinetic parameters of 7-OH CBD and the geometric averages of *T*_max_ 7-OH CBD were similar to the *T*_max_ of CBD after the administration of a single dose of CBD in a concentration range from 5–20 mg/kg, with repeated dosing for 7 days. Nevertheless, the *T*_max_ of 7-OH CBD was detected at 2 h. The geometric means of *C*_max_ of 7-OH CBD were 22.03, 34.56, and 71.7 ng/ml with the administration of a single dose of 5, 10, and 20 mg/kg of CBD, respectively. However, the *C*_max_ of 7-OH CBD increased to 2.97, 2.81, and 3.04 with repeated doses of 10, 20, and 40 mg/kg for 7 days compared with the single dose. Additionally, a decrease in the percentage of the coefficient of variation (CV%) was observed compared with a single dose. The geometric averages of AUC (0–12 h) of 7-OH CBD were 104, 202.4, and 381.9 ng h/ml with the administration of a single dose of 5, 10, and 20 mg/kg of CBD, respectively. The AUC(0-τ) of 7-OH CBD increased 4.12, 3.25, and 4.42 times with the administration of repeated doses of 10, 20, and 40 mg/kg CDB for 7 days compared with the single dose. Finally, the geometric averages of the metabolite to parent (7-OH cannabidiol/cannabidiol) ratio of AUC(0-τ) was 0.8, 0.75, and 0.76 with the administration of repeated doses of 10, 20, and 40 mg/kg for 7 days, respectively (Wheless et al., 2019).

In another study, the mean plasma concentrations of CBD and its metabolites 6-OH-CBD and 7-COOH-CBD were detected after the administration of a single dose (1.25 mg/kg) or multiple doses (5, 10, or 20 mg/kg/day) of CBD. The most abundant circulating metabolite was 7-COOH-CBD. At the end of the treatment, the AUC(0-τ) of 7-COOH-CBD was 13–17 times higher than the AUC(0-τ) of CBD. The CV% was considered to be high to moderate (20–121%; Devinsky et al., 2018b).

#### Elimination

The geometric averages of the apparent terminal half-life (*t*_1/2_) of CBD were 26.4, 29.6, and 19.5 h with the administration of a single dose of 5, 10, and 20 mg/kg, respectively. The geometric averages of the apparent total body clearance after oral administration at steady state (CLss/F) were 9.9, 12.3, and 9.5 L/h/kg, with the administration of repeated doses of 10, 20, and 40 mg/kg for 7 days, respectively (Wheless et al., 2019). Regarding the pharmacokinetic parameters of its metabolite 7-OH CBD, the geometric means of *t*_1/2_ were 18.4, 25.6, and 14.2 h with the administration of a single dose of 5, 10, and 20 mg/kg, respectively (Wheless et al., 2019). The metabolites of 7-OH-CBD are excreted in the feces and, to a lesser extent, the urine (Ohlsson et al., 1986).

During interactions with other drugs, the pharmacokinetic parameters of CBD change significantly with the administration of clobazam (CBZ). Therefore, it is often necessary to reduce clobazam due to excessive sedation. Pediatric patients who received repeated doses of 40 mg/kg for 7 days of CBD and CBZ had a 2.36-fold increase in CBD AUC(0-τ) and a 2.5-fold increase in CL/F compared with those who did not receive CBZ (Wheless et al., 2019). Conversely, CBD inhibits CYP2C19, and CYP3A4, which catalyze the metabolism of norclobazam (nCBZ). nCBZ is detected at high plasma concentrations (500% 300% mean increase) compared with the increase detected in CBZ (60% 80%) in a concentration range from 20–25 mg/kg/day of CBD and 0.18–2.24 mg/kg/day of CBZ (Geffrey et al., 2015).

### Clinical Evidence of Cannabinoids in the Treatment of Pediatric Epilepsy

Clinical studies reported below assess the safety and/or efficacy of CBD in addition to common AEDs. Most of these studies enrolled pediatric patients (0.5–17 years) with diagnoses of genetically based epilepsy, Dravet syndrome, Lennox Gastaut, febrile seizures, and seizures associated with tuberous sclerosis are the most difficult epilepsies to treat, and the latest generation of antiepileptic drugs offers limited control for these diseases (Devinsky et al., 2017; Lattanzi et al., 2018, 2019). The use of *CS* in an empirical way has been documented for a long time for the treatment of epilepsy, and in most cases, reports provide a subjective perception of benefit. Despite the positive effects of the use of medicinal cannabis CBD for the control of epileptic seizures, there is controversy in the use of CBD at the pediatric level. The first trials examining purified CBD (Epidiolex) were launched as an expanded access program (EAP) in 2014 for patients with refractory epilepsy. Szaflarski et al. (2018) published provisional data for 600 patients who used CBD during 96 weeks, revealing a reduction of seizure events by 51% mainly in cases related to Dravet syndrome, Lennox Gastaut, and tuberous sclerosis complex (reviewed by Silvestro et al., 2019).

Given the increase in the use of handcrafted cannabinoids in pediatric epilepsy and the lack of studies providing data on their safety and usefulness, a prospective investigation was carried out in 32 children with refractory epilepsy. CBD was administered at a dose of 10 mg/kg/day for 12 weeks, and the frequency of seizures and serum CBD levels were evaluated. There was a 50% reduction in the frequency of seizures during the final weeks compared with the initial ones (Knupp et al., 2019). In another open comparative study on the use of CBD to treat epilepsy, 55 patients aged 1 and 30 years with CDKL5 deficiency disorder and Aicardi, Doose, and Dup15q syndromes were included. The patients were treated in 11 institutions from January 2014 to December 2016, and a decrease in the frequency of seizures was reported from the start of CBD treatment. The dose used ranged between 10 and 20 mg/kg/day orally, and this open-label trial provided class III evidence for the long-term safety and efficacy of CBD administration in patients with refractory epilepsy (Devinsky et al., 2014, 2016, 2018a,b; Elliott et al., 2018).

In a clinical trial of children aged 1–17 years with refractory epilepsy (60), an oral solution of CBD at doses between 5, 10, and 20 mg/kg/day was administered for 12 weeks as a complement to their regimen of antiepileptic drugs. A 50% decrease in seizure frequency was observed, no serious adverse effects were found, and CBD was well tolerated (Wheless et al., 2019). In most trials, CBD is administered orally as an oil solution. In open trials, maximum doses of 25 mg/kg/day have been used in controlled studies, and higher doses of up to 50 mg/kg/day were also used; however, studies on Lennox Gastaut syndrome have shown that a significant proportion of children respond to doses of 10 mg/kg/day. Therefore, a “slow start” and “scale as appropriate” strategy is recommended, beginning with 5 mg/kg/day, increasing to 10 mg/kg/day after 2 weeks, reviewing the clinical response and adverse effects, remaining with the dose if it is effective, and otherwise increasing the dose of 5 mg/kg/day according to tolerance up to a maximum of 20–25 mg/kg/day (Treat et al., 2017; Devinsky et al., 2018b; Arzimanoglou et al., 2020).

In the previous systematic review, published in August 2017, examined 22 clinical trials, only five of which were controlled clinical trials that included a total of 795 children. The greatest evidence was for its use as an antiemetic, analgesic, and antiepileptic agent (Wong and Wilens, 2017). Currently, only two synthetic products derived from cannabis are approved by the FDA of the United States: dronabinol and nabilone. Both contain Δ^9^–THC as the main cannabinoid and are specified for the treatment of cancer comorbidities and anorexia associated with patients with HIV/AIDS (Pertwee and Cascio, 2014; Wong and Wilens, 2017).

To date, in Uruguay, the only pharmaceutical specialty registered by the MS is epifatan as 2% or 5% oral solution (drops), which contains 2 g or 5 g of CBD, respectively, and less than 0.1% THC and tetrahydrocannabinolic acid (THCA) every 100 ml. Most reports concerning the therapeutic effect of CBD in drug-resistant epilepsy have been conducted in children and adolescents (Wong and Wilens, 2017), thus providing the greatest amount of available scientific evidence. In recent decades, various investigations have been carried out on the use of cannabis in the treatment of refractory epilepsy in children, especially in epileptic syndromes such as DS, Doose, and LGS (Wong and Wilens, 2017).

In the same way, Tzadok et al. (2016) in a retrospective review of a cohort of 74 children and adolescents with drug-resistant epilepsy reported that CBD reduced the frequency of seizures in 89% of patients. In a survey of 19 parents of children with treatment-resistant epilepsy, Porter and Jacobson (2013) found that CBD treatment of 117 children with drug-resistant epilepsy reduced the seizure frequency in 84% of the patients. Conversely, in a case series of six children with drug-resistant epilepsy, dronabinol has also been reported to reduce seizures in two patients. However, most of the studies have shown inconsistency in terms of the control of the variables and absence of a placebo group, making it difficult to generate an accurate conclusion, in addition to secondary effects such as drowsiness, diarrhea, and decreased appetite (Tzadok et al., 2016). Additionally, in controlled trials, Δ^9^THC most commonly led to side effects of drowsiness and dizziness, with greater severity associated with higher doses (Wang et al., 2008). Likewise, cannabis overdose has been reported with multiple adverse effects, among which are reports of seizures among young children, which may be due to the toxicity of high-dose Δ^9^ THC (Wang et al., 2008; Mechoulam and Parker, 2013).

However, clinical investigations have methodological limitations, such as the small population of children included, the short follow-up period, and the methodological design. Nevertheless, they have reported a reduction between 57% and 84% in the frequency and intensity of epileptic seizures, with a greater impact on DS and LGS (Wong and Wilens, 2017). Few studies have examined medical cannabinoids for the treatment of seizures in children and adolescents, and they have reported that CBD reduces the frequency of seizures in the pediatric population with resistance to initial treatment in epilepsy of different etiologies (Devinsky et al., 2017; Huntsman et al., 2020). Additionally, since 2014, CBD has been administered in a continuously EAP. In a review of 119 pediatric epilepsy patients, Treat et al. (2017) reported that oral CS extracts improved seizures in 49% of the cohort. In a second study, the oral CS extracts reduced the frequency and intensity of seizures in 57% of 75 patients with drug-resistant epilepsy (Treat et al., 2017).

In addition to the lack of studies on CS, the long-term risks associated with medical CBD in pediatric patients limits our understanding of the mechanism of action and its secondary effects on neurodevelopment and epileptogenesis (Mechoulam and Parker, 2013). However, evidence has shown that onset of CS use before the age of 16 years has a relationship with cognitive and verbal learning deficits and poor psychomotor performance and attention, similar to later onset of CS, which have been associated with poorer attention, executive functioning, memory and verbal performance (Solowij et al., 2011). Similarly, frequent and recreational CS use before the age of 15 years is associated with an increased risk of depression, confusion, and subsequent suicidal tendencies. In contrast, cannabis use in early adolescence shows a greater relationship with the early onset of psychotic disorders (Hayatbakhsh et al., 2007; Claudet et al., 2017). When using CBD as an adjuvant with other antiepileptic drugs, there was an increase in liver enzymes, jaundice, and thrombocytopenia, but these alterations were resolved when the antiepileptic was discontinued. An interaction is observed between the use of CBD and clobazam, and the main adverse reactions to the use of CBD are fatigue and diarrhea; however, an increase in appetite has been documented in some patients (Geffrey et al., 2015). There is evidence of the efficacy of a large percentage of CBD in epilepsy of different etiologies despite the few studies conducted to date. However, some of them have a risk of bias or poor follow-up by caregivers of the patients. Therefore, more protocols or experimental studies are suggested with internationally approved administration doses according to age, weight, and specific indication. Such studies will provide a broader picture to not only test the efficacy of drugs in epilepsy but also study the adverse effects of drugs derived from cannabis.

## Conclusions

Evidence describing the effects of the major cannabinoids that do not act as CBR ligands, in particular cannabidiol and cannabidivarin, reveals consistently beneficial therapeutic effects in preclinical models of seizures, epilepsy, epileptogenesis, and neuroprotection. The emerging results of clinical trials in humans have methodological limitations, such as the small population of children included, the short follow-up period, and the methodological design, although, in most of them, they have reported a reduction in the frequency and intensity of seizures, in the drug-resistant pediatric population of different etiologies. What is clear is that the beneficial effects in epilepsy for the immature brain vary compared to the adult brain, due to neurodevelopmental processes. Therefore, more protocols or experimental studies should be performed with a stricter, broader, internationally approved administration dose according to age, weight, and specific indication.

## Ethics Statement

All procedures, including the use, management and care of animals, adhered to the guidelines of and were approved by the Ethics and Research Committee of the Hospital of Specialties in the National Medical Center Century XXI, Mexican Social Security Institute (IMSS) and Mexican Official Standard [1999]; NOM-062-ZOO, 1999.

## Author Contributions

AV-G developed the topic of epileptogenesis, performed the experiments with kainic acid and evaluated the latencies with the support of AM-M. IF-R, AM-A, and EZ-M developed the clinical studies. AG-J reviewed the derivatives of Cannabis and participated in the images. EG-A developed with AV-G the effects of CD on neurodevelopment. SO-S developed the idea, the topics to be considered, organized and revised the manuscript. All authors contributed to the article and approved the submitted version.

## Conflict of Interest

The authors declare that the research was conducted in the absence of any commercial or financial relationships that could be construed as a potential conflict of interest.

## Abbreviations

CNS: central nervous system
ABHD6: α-β-hydrolase domain 6
ACEA: arachidonyl-2^′^-chloroethylamide
AEA: anandamide
eCBS: endocannabinoid system
eCB-endocannabinoide CBD: cannabidiol
CBDV: cannabidivarin
CB1R: cannabinoid type 1 receptor
CB2R: cannabinoid type 2
GluR: glutamate receptor
DAGL: diacylglycerol lipase
Δ^9^-THC: Δ^9^-tetrahydrocannabinol
DSE: depolarization-induced suppression of excitation
DSI: depolarization-induced suppression of inhibition
FAAH: fatty acid amide hydrolase
GABA γ: aminobutyric acid
GAD: glutamate decarboxylase
GPR: G- protein-coupled receptor
KA: kainic acid
MAGL: monoacylglycerol lipase
MDA: maximal dentate activation
MES: maximal electroshock
NAPE-PLD: N-acylphosphatidylethanolamine-hydrolyzing phospholipase D
PMSF: phenylmethane sulfonyl fluoride
PTZ: pentylenetetrazole
TRPV1: transient receptor potential vanilloid receptor (type 1)
NMDArs: N-methyl-d-aspartate receptor
AMPArs: AMPA-type glutamate receptors
FS: febrile seizures
SE: *Status epilepticus*
TBI: traumatic brain injury
PN: post natal
eCB: endocannabinoid system
CCK: colecistoquina
Ca^2+^: calcium
K^+^: potassium
Cl^−^: chlorine
Cox-2: cyclooxigenase 2
2-AG: 2-arachidonoylglycerol
AA: arachidonic acid
AEA: N-arachidonoyl-ethanolamine or anandamide
CNS: central nervous system
Cox: cyclooxygenase
IL-1β: interleukin-1β
LOX: lipoxygenase.
